# ^13^C Natural Abundance of Serum Retinol Is a Novel Biomarker for Evaluating Provitamin A Carotenoid-Biofortified Maize Consumption in Male Mongolian Gerbils^1^,^2^,^3^

**DOI:** 10.3945/jn.116.230300

**Published:** 2016-06-08

**Authors:** Bryan M Gannon, India Pungarcher, Luciana Mourao, Christopher R Davis, Philipp Simon, Kevin V Pixley, Sherry A Tanumihardjo

**Affiliations:** 4 Department of Nutritional Sciences, Interdepartmental Graduate Program in Nutritional Sciences,; 5 Department of Horticulture, Vegetable Crops Research Unit, and; 6 Department of Agronomy, University of Wisconsin, Madison, WI; and; 7 International Maize and Wheat Improvement Center, Texcoco, Mexico

**Keywords:** biofortification, stable carbon isotope, vitamin A+, carrot, GCCIRMS, vitamin A effectiveness, vitamin A efficacy

## Abstract

**Background:** Crops such as maize, sorghum, and millet are being biofortified with provitamin A carotenoids to ensure adequate vitamin A (VA) intakes. VA assessment can be challenging because serum retinol concentrations are homeostatically controlled and more sensitive techniques are resource-intensive.

**Objectives:** We investigated changes in serum retinol relative differences of isotope amount ratios of ^13^C/^12^C (δ^13^C) caused by natural ^13^C fractionation in C_3_ compared with C_4_ plants as a biomarker to detect provitamin A efficacy from biofortified (orange) maize and high-carotene carrots.

**Methods:** The design was a 2 × 2 × 2 maize (orange compared with white) by carrot (orange compared with white) by a VA fortificant (VA+ compared with VA−) in weanling male Mongolian gerbils (*n* = 55), which included a 14-d VA depletion period and a 62-d treatment period (1 baseline and 8 treatment groups; *n* = 5−7/group). Liver VA and serum retinol were quantified, purified by HPLC, and analyzed by GC combustion isotope ratio mass spectrometry for ^13^C.

**Results:** Treatments affected liver VA concentrations (0.048 ± 0.039 to 0.79 ± 0.24 μmol/g; *P* < 0.0001) but not overall serum retinol concentrations (1.38 ± 0.22 μmol/L). Serum retinol and liver VA δ^13^C were significantly correlated (*R*^2^ = 0.92; *P* < 0.0001). Serum retinol δ^13^C differentiated control groups that consumed white maize and white carrots (−27.1 ± 1.2 δ^13^C‰) from treated groups that consumed orange maize and white carrots (−21.6 ± 1.4 δ^13^C‰ *P* < 0.0001) and white maize and orange carrots (−30.6 ± 0.7 δ^13^C‰ *P* < 0.0001). A prediction model demonstrated the relative contribution of orange maize to total dietary VA for groups that consumed VA from mixed sources.

**Conclusions:** Provitamin A efficacy and quantitative estimation of the relative contribution to dietary VA were demonstrated with the use of serum retinol δ^13^C. This method could be used for maize efficacy or effectiveness studies and with other C_4_ crops biofortified with provitamin A carotenoids (e.g., millet, sorghum). Advantages include no extrinsic tracer dose, 1 blood sample, and higher sensitivity than serum retinol concentrations alone.

## Introduction

Biofortifying staple and horticultural foods with provitamin A carotenoids can sustainably ensure adequate vitamin A (VA)^8^ intakes (1) and mitigate potential hypervitaminosis A risks caused by preformed VA in high-dose supplements and fortified foods (2–4). The bioefficacy of high provitamin A (orange) maize, defined as the production of retinol from consumed provitamin A carotenoids (5), has been demonstrated in gerbil studies (6, 7) and single-meal feeding studies in humans (8, 9). To evaluate health-promoting interventions in humans, efficacy and effectiveness trials are conducted. Efficacy trials are characterized by ideal circumstances that maximize the likelihood of observing a treatment effect: a selected homogeneous population, standardized intervention, and experienced providers or study facilitators. Effectiveness trials are characterized by real-world circumstances designed to determine whether the intervention works as actually used or adopted: a broad heterogeneous population, less standardized treatment protocols, and usual providers (10, 11). A human bioefficacy study determined that orange maize (OM) is an efficacious VA source in children (3), but to our knowledge effectiveness trials have not yet been carried out.

Liver VA concentration is the gold standard for evaluating VA status (12); however, this is only feasible in animal studies or in special cases in humans. Serum retinol concentrations are homeostatically controlled over a wide range of liver reserves (12–14), are affected by infection and inflammation (15, 16), and are nonsensitive indicators of changes in VA status (12). Furthermore, several indicators used for VA assessment, such as serum retinol and dose-response tests, are qualitative because they only distinguish deficiency from adequacy. Studies performed for provitamin A-biofortified maize in humans have used multiple blood draws for postprandial response (9) or stable isotope methods with intrinsically labeled maize (8) and tracer VA doses for isotope dilution (3). These techniques require dosing and multiple blood samples per subject, which may not be practical in large-scale effectiveness studies, particularly in children.

Most plants used for food, including staples such as wheat and rice, are C_3_ plants; however, there are a few notable crops used for human consumption that are C_4_ plants (e.g., maize, millet, sorghum, sugar cane) (17). C_3_ plants discriminate more against ^13^C during photosynthesis and therefore have lower ^13^C enrichment than C_4_ plants (18). ^13^C content at natural abundance concentrations is often expressed using the δ notation, which refers to Vienna Pee Dee Beleminite (VPDB) and is expressed as δ^13^C = [*R*_sample_/*R*_VPDB_] − 1; *r* = ^13^C/^12^C (19). This value is then typically expressed per mil (‰) by multiplying by 1000. VPDB is relatively enriched compared to most natural materials and has been assigned a δ^13^C value of 0; therefore, most other natural materials have negative δ^13^C values. Atmospheric CO_2_ is relatively stable geographically and topographically and has reported δ^13^C values ranging from −7.4‰ to −6.7‰ (20). C_4_ plants typically have δ^13^C values closer to atmospheric CO_2_ [e.g., maize (−13.6‰ and −14.0‰) (18), sorghum (−13.8‰ and −14.4‰) (18), and millet (−10.7‰ and −12.0‰) (21)]. C_3_ plants have lower δ^13^C [e.g., carrots (29.5‰ ± 0.2‰), bananas (−26.6‰ ± 0.1‰), and mangos (−25.4‰ ± 0.1‰) (17)]. Lipids and other secondary metabolites (including carotenoids) are further reduced in δ^13^C by ∼5−10‰ (22, 23); however, the difference between C_3_ and C_4_ plants is maintained, as noted with lutein obtained from marigold compared with maize (−29.9‰ ± 0.2‰ and −19.8‰ ± 0.3‰, respectively) (23). This difference gives the potential to in vivo metabolites in determining dietary plant origins based on ^13^C composition. Because no carbon is gained or lost during the cleavage of β-carotene or other provitamin A carotenoids to VA (24), the δ^13^C of serum retinol can reflect the dietary sources, including preformed and provitamin A (25). The principle of isotope mass balance states that the amount of heavy isotope in a system is a linear combination of its components (19), which could be used to quantitatively estimate the relative contributions of dietary vitamin A sources.

Several C_4_ crops are being biofortified with provitamin A carotenoids; in addition to maize, sorghum (26) and millet (27) are also targets. C_4_ plants often have advantages over C_3_ plants under conditions of drought, heat, and CO_2_ or nitrogen limitations, and for this reason they are major crops in tropical and subtropical regions (28). Furthermore, they may play a vital role in food and nutrition security under changing climates (28, 29). These biofortified varieties should be confirmed for VA bioefficacy and effectiveness at the population level.

This controlled study was undertaken to determine whether β-carotene efficacy from OM could be demonstrated with the use of shifts in the δ^13^C of serum retinol from the natural enrichment of maize feeding and comparing these values to the δ^13^C of liver VA, liver VA concentrations, and serum retinol concentrations. The δ^13^C was determined with GC combustion isotope ratio MS, which is known for its high degree of precision at natural abundance concentrations (30). Mongolian gerbils are a useful model for human absorption and metabolism of provitamin A carotenoids (31–33). Findings in maize could also extend to millet and sorghum because of their similar ^13^C enrichment.

## Methods

### 

#### Maize.

The biofortified OM was developed at the International Maize and Wheat Improvement Center in Mexico as part of its HarvestPlus biofortified maize research project (34). Seed was shipped from Mexico to Zambia, and the grain of this OM variety was produced on a commercial farm in Central Province, Zambia.

Orange maize was stored frozen (−20°C to −10°C) after harvest. White maize (WM) is a locally consumed variety in Zambia. Both varieties were hand-carried to the University of Wisconsin to be used in this study.

#### Carrots.

Carrots from the USDA carrot breeding and genetics program were grown by the University of California Desert Research and Extension Station in sandy loam soil in October and harvested in March. Carrots were refrigerated at 2°C until shipped overnight from California to Wisconsin. Upon arrival, they were returned to 2°C until freeze-dried for feed preparation. The genotypes used (i.e., high carotene mass and B2327) were selected for high β-carotene concentrations.

#### Gerbil feed.

Gerbil feeds were formulated with assistance from Harlan-Teklad to meet National Research Council-recommended macro- and micronutrient needs (35). Feeds were 50% maize by weight (36) and were modified by adding carrots at 1.5% by weight (**Supplemental Table 1**). Maize, carrots, or the VA fortificant provided the sources of VA. The retinyl palmitate used as the fortificant was dry vitamin A palmitate (250,000 IU VA/g; DSM Nutritional Products Ltd.) and was added at a concentration to meet ∼50% of estimated utilization rates found in previous studies [2.7 − 5.1 μg retinol activity equivalents (RAEs)/100 g body weight (33, 37)], resulting in a target concentration of 0.25 μg RAE/g feed. All other feed constituents were constant between groups. Treatment groups were differentiated by 3 factors: OM compared with WM, orange carrots (OCs) compared with white carrots (WCs), and VA fortificant (VA+ compared with VA−) in a 2 × 2 × 2 factorial design.

#### Gerbils and study design.

Gerbils for this study were a random subset of a larger gerbil feeding study (*n* = 85). Male 28-d-old Mongolian gerbils (Charles River Laboratories) were group housed during VA depletion (2–3/cage) and treatment (2/cage). Animal handling procedures were approved by the University of Wisconsin College of Agricultural and Life Sciences Animal Care and Use Committee. Gerbils were weighed daily for 2.5 wk and thereafter 3 times/wk. Room temperature and humidity were held constant with a 12-h light/dark cycle. Gerbils consumed ad libitum. During the depletion period (days 1–14), all gerbils consumed WM, WCs, and VA− feed. After 14 d, a baseline group kill (*n* = 5) was performed by exsanguination while the gerbils were under isoflurane anesthesia. The remaining gerbils were weight matched and allocated into 8 groups (*n* = 5–7/group) for the treatment period (days 15–77). After a 62-d treatment period (day 77), a final kill (*n* = 50) was performed as described previously.

#### Carotenoid and retinoid analyses.

All sample analyses for carotenoids and retinoids were performed under gold fluorescent lights to prevent photo-oxidation and isomerization. Feeds were analyzed for carotenoids by a published procedure for extraction (38) and an HPLC system (36). Feeds were analyzed for retinol with the same extraction and a minor modification of the HPLC system for retinol (39); solvent A was acetonitrile:water (92.5:7.5, vol:vol), and solvent B was acetonitrile:methanol:dichloroethane (80:10:10, vol:vol), both with triethylamine (0.05%, vol:vol). Serum retinol was extracted with a modified published procedure (40). Briefly, ∼1 mL serum, 1 mL ethanol, and 25 μL C23 β*-*apocarotenol in methanol were extracted twice with 1.5-mL hexanes, dried under nitrogen, resuspended in 80 μL methanol, and injected onto the first HPLC system for quantification and primary purification (3). Liver retinol and retinyl esters were analyzed by a modified published procedure (39); retinol and retinyl esters were summed to report total VA. Modifications included using ∼0.5 g liver and C23 β-apocarotenol as the internal standard, resuspending the dried aliquot in 100 μL methanol:dichloroethane (75:25, vol:vol), and using a 25-μL injection volume of reconstituted sample for HPLC.

#### ^13^C determinations.

Serum retinol was further processed for ^13^C content by a published procedure (3), including an additional HPLC purification step, drying under vacuum centrifugation, resuspension into hexanes, and injection onto the GC combustion isotope ratio mass MS system (**Supplemental Figure 1**) (25). A separate aliquot of the liver lipid extract was saponified and extracted (7); the resulting retinol was purified and analyzed similarly to serum retinol.

Maize and carrot total carbon δ^13^C were determined using an elemental analyzer combined with isotope ratio MS (41). Retinyl acetate from the VA fortificant was saponified and analyzed similarly to liver VA. All feed samples were analyzed in triplicate.

#### Estimation of maize contribution to dietary vitamin A.

The mass balance (isotope balance) equation (19, 30) was adapted to the population level and solved for the relative contribution of maize to the total VA intake in terms of serum retinol δ^13^C‰ of the test and control groups (**Supplemental Methods**):


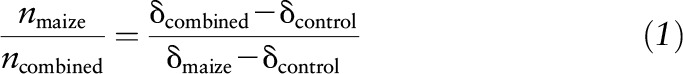


where *n* is RAEs expressed as moles and the corresponding subscripts “maize” and “combined” refer to contribution from maize and total VA, respectively. Mean serum retinol δ^13^C‰ is represented by δ, and the corresponding subscripts refer to treatment groups (control: group consuming no OM; maize: group receiving VA only from OM; combined: group consuming VA sources of control group in addition to OM). Experimental groups were fit to this model to examine whether the calculated proportion of VA coming from maize matched analytical data. WMOCVA−, WMWCVA+, and WMOCVA+ were used as 3 control groups; OMOCVA−, OMWCVA+, and OMOCVA+ were used as the 3 respective test groups that consumed combined sources; and OMWCVA− was used as the maize-only group. A bioconversion of 12 μg β-carotene equivalents:μg RAEs was used (12).

#### Statistical analysis.

Values are reported as means ± SDs. Data were analyzed with the use of SAS version 9.4. Outcomes of interest were evaluated with the use of independent 2-sample, 2-tailed *t* tests or 3- and 1-factor ANOVA to compare treatment groups and to determine differences between groups with the use of the general linear model procedure as appropriate. Feeds were compared with the use of 1-factor ANOVA. Linear regression was also performed with the general linear model procedure. Post hoc letter groupings between treatment groups were determined with the use of least significant differences. Normality of residuals was tested with the Shapiro-Wilk test; homogeneity of variance was tested with Levene’s test. Data failing normality or variance assumptions were analyzed nonparametrically by analyzing ranked data. *P* < 0.05 was considered significant.

## Results

### 

#### Feed properties.

Carotenoid and retinol equivalent concentrations in the feeds had the expected relations (**Table 1**). OM provitamin A was predominantly β-carotene (∼96%) with some β-cryptoxanthin (∼3%) and α-carotene (∼1%). Carrot provitamin A was mostly β-carotene (∼65%) but with appreciable α-carotene (∼34%) and minimal β-cryptoxanthin (∼1%). Maize total carbon δ^13^C was higher than carrots for both OM (−11.0‰ ± 0.2‰ compared with −25.6‰ ± 0.2‰ *P* < 0.0001) and WM (−11.3‰ ± 0.3‰ compared with −25.3‰ ± 0.1‰ *P* < 0.0001) varieties; δ^13^C did not differ within carrot or maize varieties (*P* ≥ 0.05). The preformed retinyl palmitate used as the fortificant had a δ^13^C of −27.4‰ ± 1.2‰, which represents only the retinol portion because the sample was saponified before analysis.

**TABLE 1:** Feed concentrations of provitamin A carotenoids and preformed VA in maize treatments fed to Mongolian gerbils^1^

	WCWMVA−	OMWCVA−	WCWMVA+	OMWCVA+	OCWMVA−	OCOMVA−	OCWMVA+	OCOMVA+	*P*
β-Carotene,^2^ μg/g feed	0.0111 ± 0.0037^d^	6.05 ± 0.52^c^	0.0125 ± 0.0043^d^	7.03 ± 0.26^c^	13.7 ± 0.4^b^	20.5 ± 0.4^a^	13.3 ± 0.2^b^	21.3 ± 0.9^a^	<0.0001
Preformed VA,^3^ μg RAE/g feed	ND	ND	0.370 ± 0.116	0.198 ± 0.007	ND	ND	0.188 ± 0.034	0.268 ± 0.156	
Total,^4^ μg RAE/g feed	0.000924 ± 0.000304	0.505 ± 0.043	0.371 ± 0.116	0.784 ± 0.023	1.14 ± 0.04	1.70 ± 0.04	1.35 ± 0.12	2.05 ± 0.17	—
Maize RAEs/total RAEs^5^	0.536	0.999	0.002	0.685	0.000	0.326	0.000	0.284	—

1 Values are means ± SDs, *n* = 3/feed. Labeled means in a row without a common superscript letter differ, *P* < 0.05. ND, not detected; OC, orange carrot; OM, orange maize; RAE, retinol activity equivalent; VA, vitamin A; WC, white carrot; WM, white maize.

2 β-Carotene equivalents in mass were determined by summing [β-carotene isomers + 0.5 × α-carotene + 0.5 × β-cryptoxanthin × 536.9 (molar mass β-carotene)/552.8 (molar mass β-cryptoxanthin)].

3 VA was not detected in feeds in which VA fortificant was omitted; limit of detection: 0.004 μg RAE/g feed. *P* value reflects VA+ feeds only. Target concentration: 0.25 μg RAE/g feed; overall mean ± SD: 0.25 ± 0.11 μg RAE/g feed.

4 Total RAEs were calculated using the bioconversion factor 12 μg β-carotene:μg RAEs (12).

5 Proportion of VA from maize calculated using overall mean for fortificant VA and also used the bioconversion factor 12 μg β-carotene:μg RAEs (12).

#### Serum retinol and liver VA concentrations.

**FIGURE 1:**
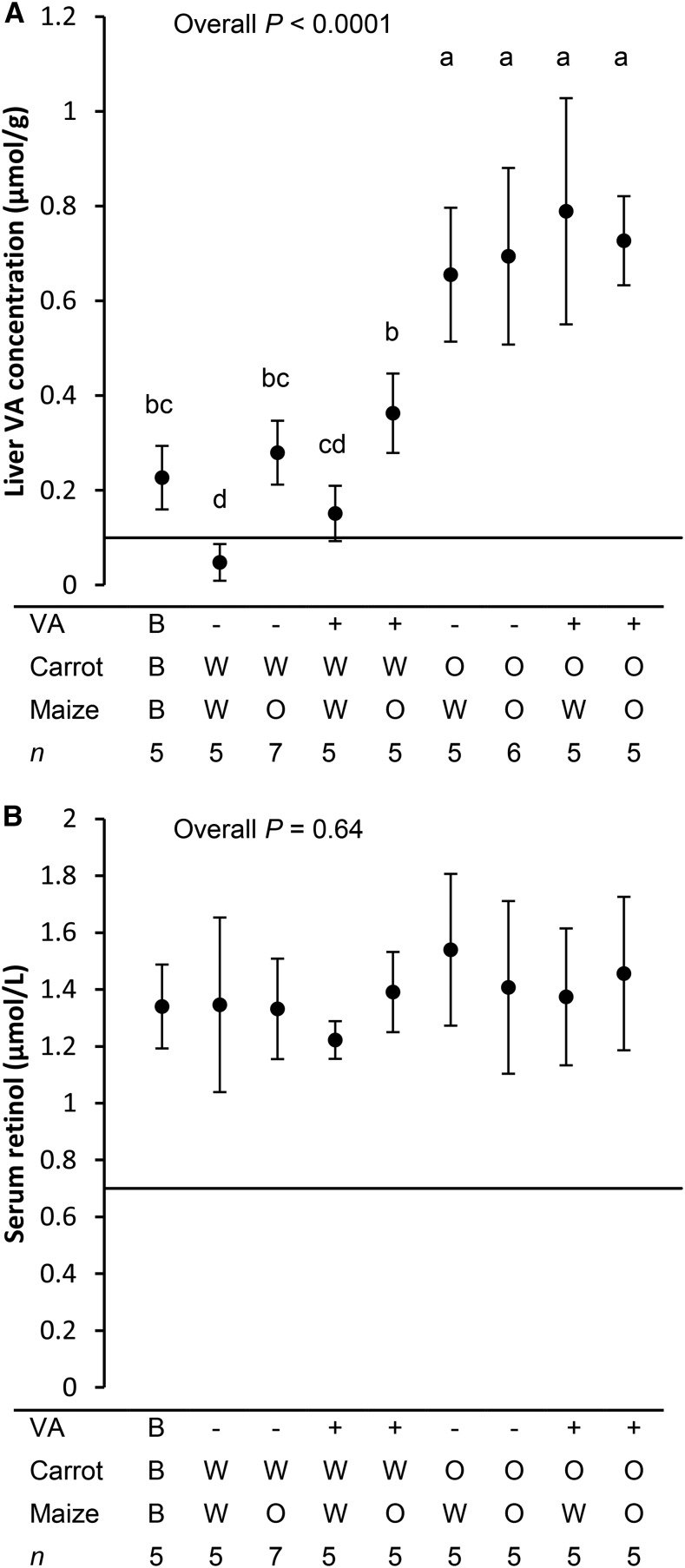
Liver VA (A) and serum retinol (B) concentrations in gerbils after consuming feeds with different combinations of provitamin A carotenoid and preformed VA sources. All values are means ± SDs (*n* = 5–7). Liver VA residuals were not normally distributed, and variance was not homogeneous; data were analyzed nonparametrically. Horizontal lines at 0.1 μmol/g (A) and 0.7 μmol/L (B) are the deficiency cutoff concentrations. Labeled means without a common letter differ, *P* < 0.05. B, baseline; O, orange; VA, vitamin A; W, white.

Serum retinol and liver VA (retinol + saponified retinyl ester) concentrations were plotted (**Figure 1A, B**). Liver VA concentrations were highly dependent on dietary VA. The group mean for the treatment group that consumed the least VA (WMWCVA−) was below the VA deficiency cutoff of 0.1 μmol VA/g liver (14) after treatment. Both provitamin A carotenoid sources increased liver VA concentrations considerably. The mean liver VA concentrations from all groups that consumed OC were not different from each other and were much greater than all groups that consumed WC. Within the WC groups, both groups that consumed OM had higher VA liver concentrations than their respective WM controls. The VA fortificant, which was meant to meet 50% of the estimated requirements of gerbils, did not notably improve VA stores. All groups that consumed the VA fortificant had liver VA concentrations that were similar to their respective controls without the VA fortificant. Nonetheless, the group that consumed appreciable VA from fortificant only (WMWCVA+) had a mean liver VA concentration >0.1 μmol/g and was not significantly different from the baseline group (*P* = 0.08), which meant they were able to maintain initial VA status. Serum retinol concentrations did not differ between groups despite a wide range of liver VA concentrations, and serum retinol was not correlated to liver VA concentrations (*R*^2^ = 0.028).

#### Serum retinol and liver VA δ ^13^C.

**FIGURE 2:**
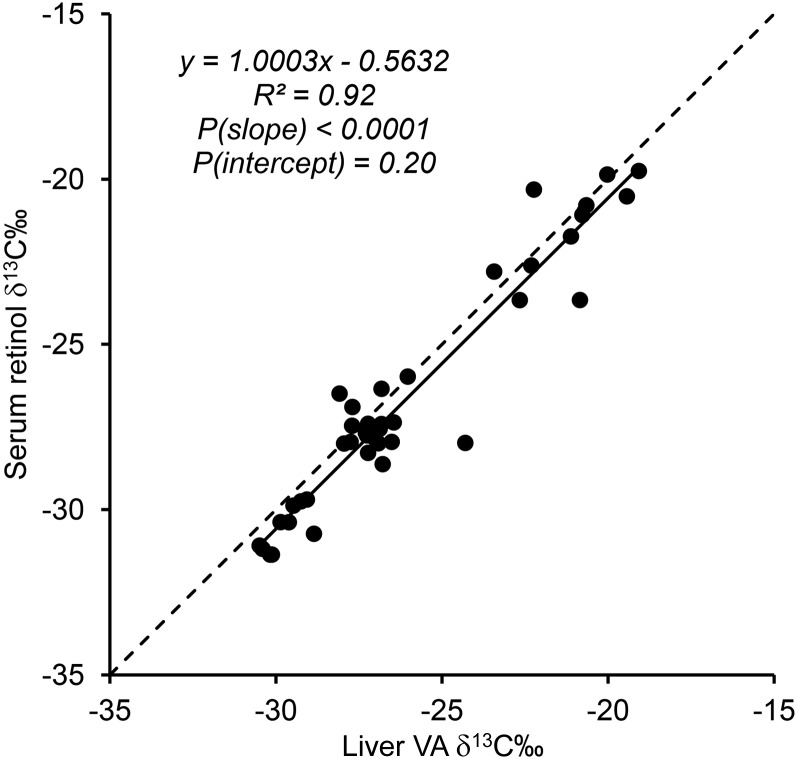
Serum retinol δ^13^C‰ plotted against liver VA δ^13^C‰ for gerbils that consumed diets with varying amounts and sources of provitamin A carotenoids and preformed VA. Only gerbils with values for both outcomes are plotted (*n* = 40; 3–7/group) along with the best fit line of the data (solid line) and *y* = *x* (dashed line). VA, vitamin A.

Serum retinol and liver VA δ^13^C had good agreement (**Figure 2**), indicating that the accessible serum retinol pool is highly reflective of the major liver VA store.

When serum retinol δ^13^C was analyzed with 3-factor ANOVA, all main effects, the VA × carrot interaction, and the maize × carrot interaction were highly significant (all *P* ≤ 0.0001). The VA × maize interaction (*P* = 0.09) and 3-factor interaction were not significant. Because of multiple interactions, 1-factor ANOVA with post hoc analysis was then used to analyze the data, including the baseline group.

**FIGURE 3:**
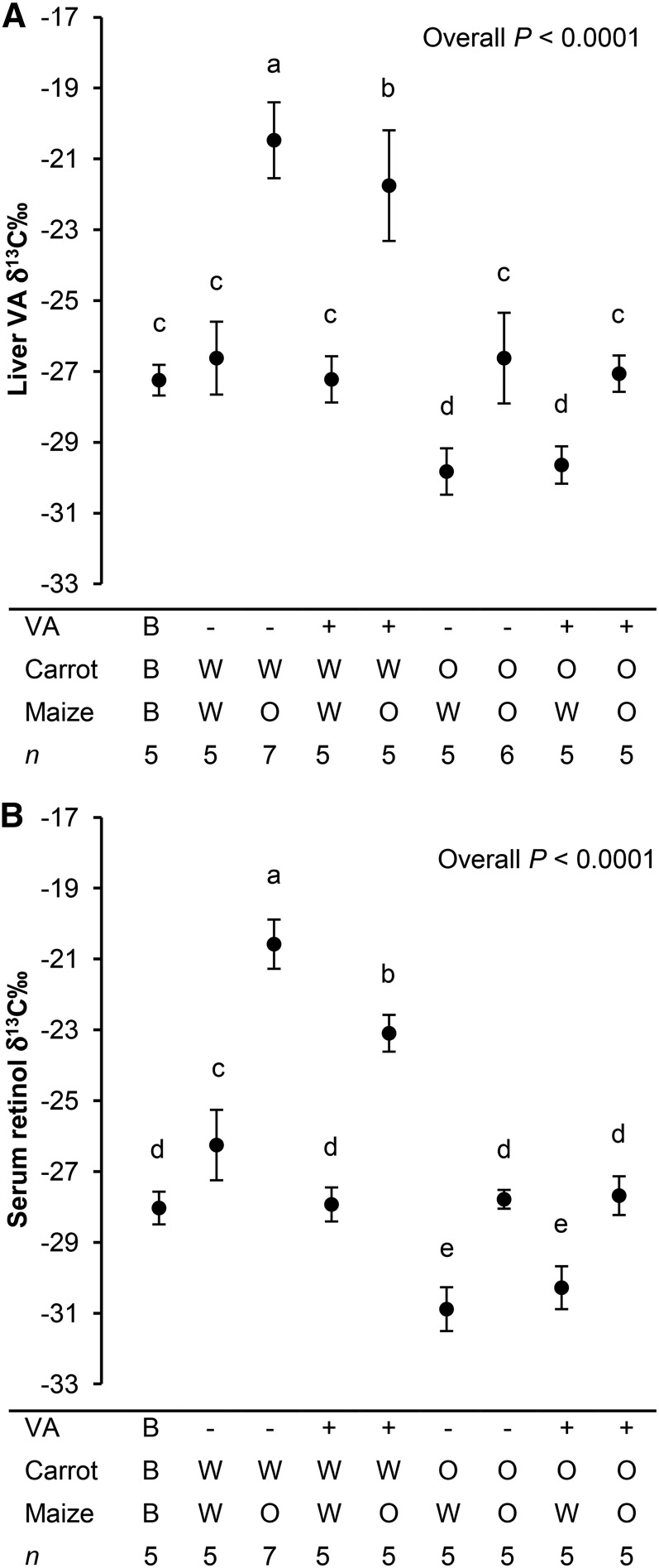
Gerbil liver VA δ^13^C‰ (A) and serum retinol δ^13^C‰ (B) after consuming feeds with different combinations of provitamin A and preformed VA sources. All values are means ± SDs. Labeled means without a common letter differ, *P* < 0.05. B, baseline; O, orange; VA, vitamin A; W, white.

Serum retinol and liver VA δ^13^C by group showed similar responses to treatment (**Figure 3A, B**). All groups that consumed OM had significantly greater VA δ^13^C than the corresponding WM controls. By contrast, all groups that consumed OCs had much lower VA δ^13^C values compared with corresponding WC controls. The group that consumed the VA fortificant as the primary VA source (WMWCVA+) had serum δ^13^C that was not significantly different than the VA fortificant (−27.9‰ ± 0.5‰ compared with −27.4‰ ± 1.2‰). The group that consumed VA almost entirely (>99.9%) from maize had a serum retinol δ^13^C of −20.6‰ ± 0.7‰.

#### Estimation of maize contribution to dietary VA.

**FIGURE 4:**
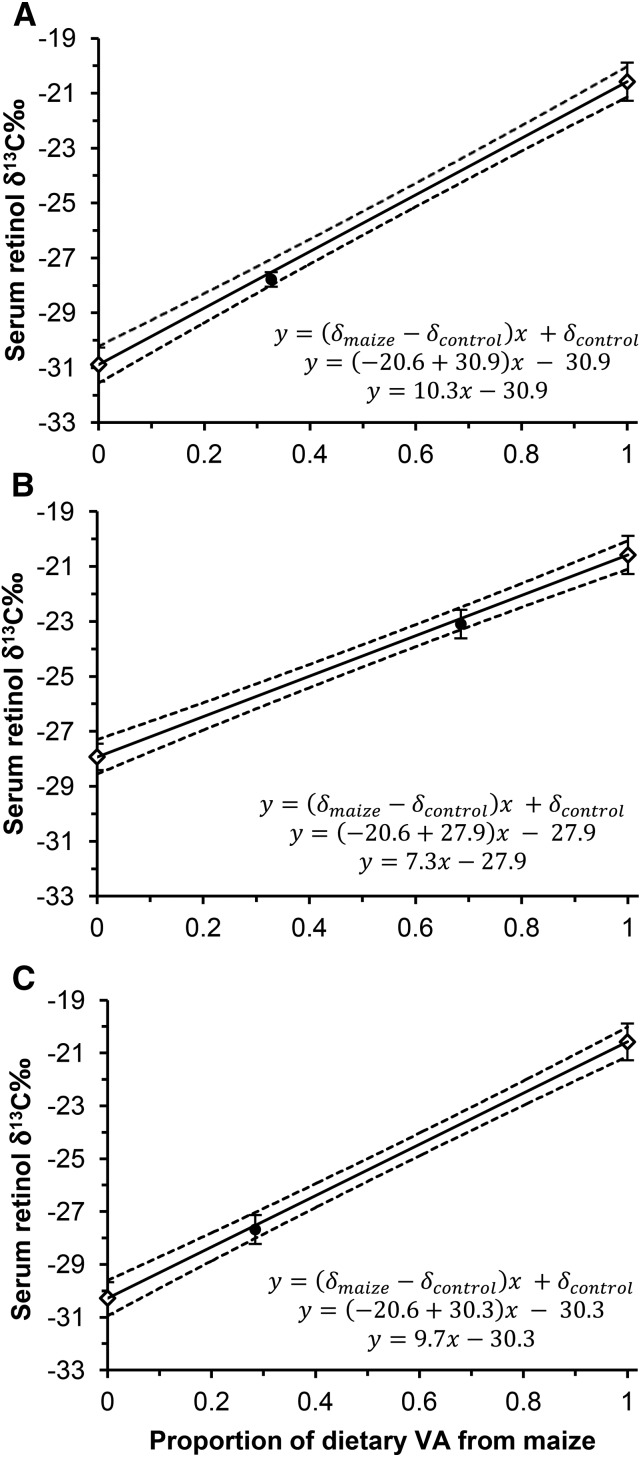
Gerbil serum retinol δ^13^C‰ plotted against the proportion of dietary VA coming from maize for diets containing varying amounts of VA in the form of preformed vitamin A and provitamin A carotenoids. For all plots, a prediction line with the use of mean serum retinol δ^13^C‰ from the control group that consumed negligible VA from maize and the maize group that consumed VA exclusively from maize (OMWCVA−, *n* = 7) was plotted against the analytical determination of maize contribution to dietary VA (open diamonds). Dashed lines: 95% confidence limits; error bars: SDs. The means ± SDs of the corresponding test group were plotted independently to test the predictive ability of Equation *1* (closed circles). (A) Control group: WMOCVA−, *n* = 5; test group: OMOCVA−, *n* = 6. (B) Control group: WMWCVA+, *n* = 5; test group: OMWCVA+, *n* = 5. (C) Control group: WMOCVA+, *n* = 5; test group: OMOCVA+, *n* = 5. OC, orange carrot; OM, orange maize; VA, vitamin A; WC, white carrot; WM, white maize.


Plots were made with the use of Supplemental Methods Equation *4* of 3 pairs of control and test groups (**Figure 4**). The proportion of dietary VA predicted from Supplemental Methods Equation *5* in the model agreed well with analytical values; all 3 test group means were within the 95% confidence limits. Supplemental Methods Equation *5* can be simplified to include data from the OMWCVA− group assuming the group mean adequately represents the serum retinol δ^13^C of gerbils that consumed VA almost entirely from maize (>99.9%). Therefore, the relative contribution of maize to total dietary VA in a population can be estimated by the serum retinol δ^13^C group means of the control and test (combined) groups:

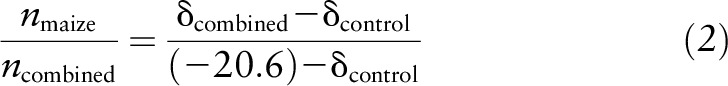


#### Experimental and analytical variation.

Experimental and analytical CVs were very low for δ^13^C and their corresponding ^13^C isotopic abundance (^13^C/total carbon) values (**Supplemental Table 2**). All δ^13^C CVs for individual experimental groups were ≤3.4%, and all ^13^C isotopic abundance CVs for individual experimental groups were ≤0.23%.

## Discussion

The analysis of δ^13^C in serum retinol allowed the determination of provitamin A efficacy from biofortified maize compared with feeds containing minimal VA, provitamin A carotenoids, preformed VA, or a combination of sources when contrasted to an appropriate control group. Furthermore, the relative contribution of maize to total VA intake was quantitatively estimated and verified against analytical determination. The first advantage is high sensitivity to detect provitamin A maize consumption because of low variation within groups that consumed the same feeds. Circulating retinol concentrations are homeostatically controlled outside of severe hypo- or hypervitaminosis A (12, 42, 43) and frequently do not respond to VA interventions (3, 14, 44, 45), both of which were observed in this study. However, they are still often used as a primary outcome to evaluate populations and interventions aimed at improving VA status (46), which may not detect the potential effects of β-carotene or VA interventions that use more sensitive methods (3, 44, 45). A second advantage is that a single blood sample after long-term consumption is required. More sensitive methods used as outcomes in VA studies, such as isotope dilution (3, 44) or appearance in serum (9), use multiple blood draws. This is undesirable—especially when working with children—and can complicate recruitment and follow-up during studies (47). Finally, no external isotopically labeled material or VA analogues, which are used for isotope dilution, tracer, and modified dose-response tests (12, 14, 48), are required. These compounds are often expensive and technically demanding to produce and prepare. Together these advantages show promise for future efficacy or large-scale effectiveness trials to evaluate crop adoption and consumption, especially in populations or settings in which resources are limited and multiple sample collections are not practical.

Variations in natural abundance ratios of stable isotopes (e.g., carbon, nitrogen, sulfur, hydrogen, oxygen) measured from numerous sources (e.g., breath, hair, nails, plasma, RBCs, and specific molecules such as alanine) have been used as biomarkers for dietary origin (49). An early study noted elevated δ^13^C in breath CO_2_ after the consumption of sugar (50), and more recent applications have further developed the methodology. For example, δ^13^C was measured from several tissues to assess sugar intake (51), and serum total carbon δ^13^C was lower after an intervention to decrease sugar-sweetened beverage intake (52). Nitrogen-15 enrichment varies between plant and animal protein sources, and this natural difference has been correlated with meat and fish intake in numerous studies (49). Reduced serum retinol δ^13^C was demonstrated in response to increased consumption of C_3_ vegetables containing provitamin A carotenoids, including carrots and pumpkin (25), which we also demonstrated in this study.

Other studies have applied GC combustion isotope ratio MS to measure per-labeled ^13^C β-carotene (53) and lutein (54) absorption, enabling the characterization of the appearance and disappearance of carotenoids in plasma after the ingestion of physiological amounts from food. Traditional MS could theoretically be used to apply our proposed method, although instead of a single CO_2_ peak with 3 mass traces from the combusted retinol to determine the ^13^C:^12^C ratio (Supplemental Figure 1), mass distributions of retinol would need to be compared, and adequate precision would first have to be demonstrated.

Although enzymatic isotope effects are established in plants and yield differences in ^13^C enrichments both in classes of metabolites (i.e., starch compared with lipid) within plants (22) and between plants exhibiting different photosynthetic systems (18), it is relatively unknown whether similar effects can be observed for VA in animals and affect organ partitioning given that numerous enzymes participate in VA metabolism (55). Excellent agreement between serum retinol and liver VA δ^13^C indicates that in a paradigm of constant long-term consumption, the use of serum retinol δ^13^C is a suitable alternative to represent that in the major liver store.

Serum retinol and liver VA δ^13^C of treatment groups agreed with data in the literature. Lipids and carotenoids are reduced in ^13^C by ∼5–10‰ compared with total carbon (22, 23), which agrees with our results that OC total carbon had a δ^13^C of −25.6‰, and the treatment group that consumed OCs as the predominant VA source (WMOCVA−) had a mean liver VA δ^13^C of −29.8‰ (a difference of 4.2‰). OM total carbon had a δ^13^C of approximately −11.0‰, and the treatment group with the only appreciable VA dietary source as OM (OMWCVA−) had a mean liver VA δ^13^C of −20.5‰ (a difference of 9.5‰). Furthermore, the liver VA δ^13^C from these 2 groups corresponded well to lutein obtained from marigold (a C_3_ plant) compared with maize (−29.9‰ and −19.8‰, respectively) (23). Serum retinol δ^13^C was similar in the gerbils that obtained VA from the fortificant only (WMWCVA+) to the δ^13^C of the fortificant itself.

Although natural ^13^C enrichment is suitable for distinguishing provitamin A carotenoid efficacy from maize compared with a WM control feed in a trial setting, this study revealed limitations on its use as a purely diagnostic tool for VA status. In addition to the baseline group, 4 treatment groups had similar serum retinol and liver VA δ^13^C despite widely varying liver VA concentrations. These treatment groups either consumed either both OM and OCs or both WM and WCs, and the resulting enrichment was a mixture of the sources. Although WM and WCs are both very low in provitamin A carotenoids, each contributed small amounts to the feed (maize: 5.2 ± 1.1 ng β-carotene/g feed; carrots: 4.5 ± 0.4 ng β-carotene/g feed), which would be reflected in the δ^13^C values. However, groups that consumed OM had considerably higher serum retinol δ^13^C and liver VA concentrations than their respective controls that consumed WM. If a population that consumed OM demonstrated elevated serum retinol δ^13^C, it could be inferred that their VA status is greater than or equal to a control population that consumed WM depending on the initial VA status of the population. δ^13^C shifts reflect the consumption of provitamin A maize but are not a replacement for evaluating VA status, such as isotope dilution methods (3). Isotope dilution or dose-response tests could be used on a subset of randomly selected individuals to confirm desired VA status in efficacy or effectiveness studies.

Liver VA concentrations were not different between all groups that consumed OCs regardless of additional VA from OM or the fortificant, likely reflecting the downregulation of provitamin A bioconversion (56, 57) and a relatively minor impact of the VA fortificant compared with OCs. Despite this, the serum retinol δ^13^C was still able to distinguish the feeds in which provitamin A carotenoids were obtained from OM or OCs. This is important considering some populations targeted for biofortification have substantial intakes of VA, even if intake varies seasonally (58, 59). Although these reports highlight a need for more sensitive markers of VA status to ensure interventions do not lead to the chronic overconsumption of VA (2, 3), biofortification of staple foods with provitamin A carotenoids can mitigate seasonal gaps in provitamin A consumption and reduce the chances of excessive preformed vitamin A intake caused by the regulation of absorption and bioconversion of provitamin A carotenoids (56, 57). A 62-d treatment period was sufficient to have serum retinol δ^13^C reflect that in the major body pool (i.e., liver) in this study, but this time requirement in humans likely depends on a number of factors, including the baseline body pool of VA, dietary VA intake, and the rate of VA metabolism. Labeled VA doses mix with body stores within 26 d after administration to adults (60) and 12 d in children (61); however, it will likely take longer for serum retinol δ^13^C to accurately reflect regular dietary consumption. If the VA pool size increases as the intervention intends, this equilibration time would be shorter.

## Footnotes

8 Abbreviations used: OC, orange carrot; OM, orange maize; RAE, retinol activity equivalent; VA, vitamin A; VPDB, Vienna Pee Dee Beleminite; WC, white carrot; WM, white maize.
